# The rph1 Gene Is a Common Contributor to the Evolution of Phosphine Resistance in Independent Field Isolates of *Rhyzopertha Dominica*

**DOI:** 10.1371/journal.pone.0031541

**Published:** 2012-02-20

**Authors:** Yosep S. Mau, Patrick J. Collins, Gregory J. Daglish, Manoj K. Nayak, Hervoika Pavic, Paul R. Ebert

**Affiliations:** 1 School of Integrative Biology, The University of Queensland, Saint Lucia, Queensland, Australia; 2 Faculty of Agriculture, the University of Nusa Cendana, Kupang, Nusa Tenggara Timur, Indonesia; 3 Department of Employment, Economic Development and Innovation, Ecosciences Precinct, Brisbane, Queensland, Australia; 4 Cooperative Research Centre for National Plant Biosecurity, Bruce, Australian Capital Territory, Australia; Kyushu Institute of Technology, Japan

## Abstract

Phosphine is the only economically viable fumigant for routine control of insect pests of stored food products, but its continued use is now threatened by the world-wide emergence of high-level resistance in key pest species. Phosphine has a unique mode of action relative to well-characterised contact pesticides. Similarly, the selective pressures that lead to resistance against field sprays differ dramatically from those encountered during fumigation. The consequences of these differences have not been investigated adequately. We determine the genetic basis of phosphine resistance in *Rhyzopertha dominica* strains collected from New South Wales and South Australia and compare this with resistance in a previously characterised strain from Queensland. The resistance levels range from 225 and 100 times the baseline response of a sensitive reference strain. Moreover, molecular and phenotypic data indicate that high-level resistance was derived independently in each of the three widely separated geographical regions. Despite the independent origins, resistance was due to two interacting genes in each instance. Furthermore, complementation analysis reveals that all three strains contain an incompletely recessive resistance allele of the autosomal *rph1* resistance gene. This is particularly noteworthy as a resistance allele at *rph1* was previously proposed to be a necessary first step in the evolution of high-level resistance. Despite the capacity of phosphine to disrupt a wide range of enzymes and biological processes, it is remarkable that the initial step in the selection of resistance is so similar in isolated outbreaks.

## Introduction

Phosphine (PH_3_) fumigation is the primary method of controlling the lesser grain borer, *Rhyzopertha dominica* (F.) as well as other highly destructive stored-grain pests. However, the emergence of resistance against phosphine in key pest species over the last three decades, threatens the future use of this critically important fumigant [1]. High level resistance in *R. dominica* has been reported from Bangladesh [2], India [3], [4], China [5], Australia [6], the Philippines [7] and Brazil [8].

Resistance to phosphine in *R. dominica* was first detected in Australia in the 1970s [9] but the resistance was considered ‘weak’, about 30 times the baseline response of a phosphine sensitive reference strain [10]. The first detection of high level resistance to phosphine in *R. dominica* in Australia was from Queensland in 1997 [6] at a level 600 times that of the sensitive reference strain [10]. Detailed genetic analysis of SR_QLD_ (elsewhwere referred to as QRD569) identified resistance alleles at two loci. The first, *rph1*, is responsible for weak resistance whereas the second, *rph2*, provides only very weak resistance on its own, but acts synergistically with *rph1* to confer high level resistance [11], [12]. This led to the proposal that high level resistance conferred by *rph2* could only arise once the resistance allele at *rph1* had already been selected. The outbreak of strongly resistant *R. dominica* in New South Wales and in South Australia [13] now lets us test whether resistance at *rph1* is a necessary component of high level resistance. The potential impact of phosphine resistance is exemplified by the Australian situation in which 80% of stored grain is protected by phosphine.

Despite the importance of phosphine, there is limited understanding of how resistance is mediated. Not only do we not understand the mechanism of resistance, but we do not even know the number of resistance mechanisms that might exist. Understanding the mechanisms behind resistance will help us not only develop tools for resistance monitoring but also fumigation strategies to forestall resistance development. Unlike the situation with field crops for which insect damage up to an economic threshold is tolerated, nil tolerance is necessary to achieve premium prices for stored grain. Nil tolerance precludes the use of refugia in resistance management and results in repeated rounds of very strong selection, coupled with severe population bottlenecks. We expect the resistance mechanisms to reflect these unique aspects of pest control in stored grain.

Whereas the selective pressures leading to resistance in a closed fumigation environment are much more strictly defined than is the case for resistance selection in field crops, the mode of action of phosphine is much broader than that of a typical contact pesticide. As a reducing agent that can interact strongly with transition metals [14], phosphine has the potential to disrupt the enzymatic activity of a large fraction of cellular proteins. Phosphine is known to disrupt mitochondrial energy metabolism leading to a decrease in ATP synthesis [15]–[17]. Phosphine also participates in the generation of toxic oxyradical species via metabolic disruption [18], release of cellular iron stores [19] and chemical interaction with hydrogen peroxide [20].

As with the mode of action of phosphine, the mechanism of resistance is unknown. Proposed hypotheses include: 1) decreased uptake of phosphine [21]–[24], 2) oxidative stress resistance 25–27, or 3) metabolic avoidance of phosphine via a decrease in reliance on oxidative respiration [28]–[31].

The present study compares the genetic basis of phosphine resistance in independent outbreaks to determine whether diverse mechanisms can lead to phosphine resistance. Specifically, we compare phosphine resistance in strongly resistant strains from New South Wales (SR_NSW_) and South Australia (SR_SA_) and determine that both contain a resistance allele at the *rph1* locus, as had previously been found in strongly resistant *R. dominica* from Queensland (SR_QLD_). This work supports the hypothesis that resistance at *rph1* is a prerequisite for the selection of strong resistance and indicates that a synergistic interaction between *rph1* and a second resistance gene is a general feature of high level resistance to phosphine. Our work indicates that despite the general reactivity and wide range of potential toxic mechanisms of phosphine [14], the number of resistance mechanisms and genes that contribute to resistance in *R. dominica* is very limited.

## Materials and Methods

### Insect strains

In total, five *R. dominica* strains were used in this study. The first two are strongly phosphine resistant and were collected in the year 1999 from Merriwagga in south-western New South Wales (NNRD2864) and in 2000 from Port Adelaide in South Australia (NSRD3075) [13]. Three other strains were collected near Millmerran in Queensland, Australia [10]. As these strains have been characterised in detail, they were used as sensitive (QRD14), weakly resistant (QRD369) and strongly resistant (QRD569) reference strains in this study. For simplicity, these strains are referred to throughout the text according to their level of resistance with their state of origin given as a subscript, thus SR_NSW_ is NNRD2864, SR_SA_ is NSRD3075, SR_QLD_ is QRD569, WR_QLD_ is QRD369 and S_QLD_ is QRD14. The approximate distance between the geographic origins of any two strongly resistant strains was 700–1500 km. All resistant strains were selected with phosphine for at least five generations to promote homozygosity. All strains were cultured on whole wheat at 30°C and 55% relative humidity. No specific permits were required for the described field collection of insects.

### Phosphine Fumigation

Responses of the parental strains and their progenies to a range of phosphine concentrations (0.001–1.5 mg/L) were examined by fumigation according to the FAO agreed standard [32] except that the fumigation time was extended from 20 hours to 48 hours [33]. Phosphine gas was generated by exposing aluminium phosphide pellets to a solution of sulphuric acid (5%) below a collecting tube [27]. Phosphine concentration was determined by gas chromatography, utilising nitrogen (N_2_) as a standard and Freon-24 as carrier gas.

Adult beetles (1–3 weeks old) were confined within plastic cups (50 beetles per cup) containing approximately 5 g of whole wheat inside gas-tight desiccators that were used as exposure chambers. Phosphine was drawn from the generation chamber [27] through a silicon septum using a gas-tight syringe and was injected into each desiccator through a septum. The insects were exposed to phosphine for 48 hours at 25°C and 55% RH. Mortality was assessed after a recovery period of 14 days at 25°C and 55% RH to ensure that end-point mortality was reached.

### Inheritance of resistance in SR_NSW_ and SR_SA_

The strongly resistant field-collected strains, SR_NSW_ and SR_SA_, were selected for high-level resistance to phosphine across multiple generations to ensure homozygosity of resistance alleles. The strains were initially exposed to 0.25 mg/L, 0.5 mg/L, or 1.0 mg/L phosphine and survivors were allowed to reproduce. Four additional rounds of selection were carried out at phosphine concentrations of 0.5 mg/L (SR_NSW_) or 0.25 mg/L (SR_SA_). With each round, survivors were allowed to reproduce, after which their progeny were exposed to the designated phosphine concentration. Following selection to homozygosity, reciprocal crosses were made between the sensitive reference strain S_QLD_ and each of the two resistant strains, SR_NSW_ and SR_SA_. To ensure the virginity of females, the insects were reared on kibbled wheat and the sex of the insects was identified at the pupal stage. The sexed pupae were placed in individual gelatine capsules containing kibbled wheat. The resulting adult insects (1–3 wk old) were paired (20 pairs per cross) and placed in plastic cups with perforated lids each containing 5 g of kibbled wheat. After 3 weeks, the cups were inspected for progeny (eggs or larvae). The parents were removed and the resulting progenies were transferred into culture bottles filled with 500 g whole grain wheat. Adults (1–3 weeks old) from the parental strains and their F_1_ progenies were fumigated as described above to determine whether the resistance trait is dominant, recessive or sex-linked.

F_2_ and F_1_ backcross (F_1_-BC) progenies were subsequently generated to test the null hypothesis that a single gene controls resistance. One hundred and fifty F_1_ individuals were allowed to mass cross for 2 weeks to produce an F_2_ generation. Virgin F_1_ females were identified at the pupal stage and were mated with their resistant male parents to produce F_1_-BC progeny.

### Complementation analysis of resistance

Complementation analysis was conducted to determine whether an allele of *rph1* contributes to resistance in the strongly resistant strains from New South Wales (SR_NSW_) and South Australia (SR_SA_). This analysis required crossing the two strongly resistant strains with a weakly resistant strain, WR_QLD_, which is homozygous for a single resistance factor, *rph1*. F_1_ and F_2_ progenies were produced from each of these crosses, and their response to phosphine exposure at a range of concentrations was assessed.

### Data analysis

All mortality data were first corrected for control mortality (≤10%) based on Abbott's formula [34] before probit analysis using log-concentration/probit-regression lines [35]. The probit analysis was carried out using the GenStat, 6 statistical package. The goodness-of-fit to the log-dose/probit mortality line was determined by a chi-square test. In the goodness-of-fit calculation, at doses where the expected response was less than one, the number of observed responses was combined with the value for an adjacent dose and the degrees of freedom for the chi-square analysis were adjusted accordingly. In the genetic study of phosphine resistance, the LC_50_ values and fiducial limits (95%) of reciprocal F_1_ crosses calculated from the regression analysis were used to determine whether the resistance was sex-linked or not. Overlapping 95% fiducial limits of reciprocal F_1_ crosses were accepted as an indication of non-significance and, hence, the absence of sex-linked inheritance of resistance. The degree of dominance in the F_1_ offspring was calculated according to the method of Stone [36] and the dominance variance was calculated according to Preisler et al. [37]. The hypothesis that a single gene is responsible for resistance was tested using F_2_ and F_1_-BC progeny response data. Two methods were employed in testing the monogenic hypothesis: Firstly, by observing the shape of the F_2_ and F_1_-BC response curves for the presence or absence of appropriate plateaus. If a single gene is responsible for resistance then a plateau is expected in the F_2_ response line at either 25 or 75% mortality, depending on whether the allele is dominant or recessive. A plateau at 50% mortality is expected in the F_1_-BC response line whether the resistance allele is dominant or recessive. Secondly, by testing the goodness-of-fit of observed and expected mortality data at individual doses using Chi-square analysis according to the following formula [38]:

- F_2_ progeny:
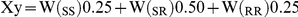

- F_1_-BC (F_1_ x resistant parent):
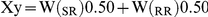
where X = the expected response at a given concentration y, W = the observed response of SS, SR, RR at concentration y, obtained from the respective regression lines. SS = homozygous sensitive parent, SR = hybrid, RR = homozygote resistant parent. The resistance factors for the parental resistant strains were calculated by dividing the LC_50_ of each parental strain by the LC_50_ of the sensitive strain. Similarly, the resistance factors for the heterozygotes (SR) were determined by crossing each resistant strain with the sensitive strain and dividing the LC_50_ of the F_1_ progeny by the LC_50_ of the sensitive strain. Data from reciprocal F_1_ crosses were pooled in calculating the resistance factor for the F_1_.

### Molecular Diagnostic of Phosphine Resistance

Molecular marker STS5.11 was used to determine whether the resistant strains employed in this study share a resistance allele at a second resistance gene. The marker is very tightly linked to a resistance locus, *rph2*, of the strongly resistant strain SR_QLD_ [12]. Genomic DNA was extracted from a single beetle from each of the five strains utilising a chelating resin, Chelex® 100 (Sigma, St Louis, MO, USA), following the method described in Schlipalius et al. [11]. The DNA of the insects was amplified by PCR using RP5.11 specific primers (Forward: 5′–TGCTGGTTACCCCAAATCAG–3′ and Reverse:5′–AGATCGCGTGGGTAACCAGCA–3′), based on the method described by Schlipalius et al. [11] with a slight modification. Each 20 µL PCR reaction contained 2 µL of 10× PCR buffer (100 mM Tris HCl pH 8.0, 100 mM KCl, 15 mM MgCl_2_), 2 µL of 1 mM dNTPs, 1 µL each of 10 µM forward and reverse primers, 1 µL of 1 U/µL REDTaq® DNA polymerase (Sigma), 1 µL DNA template and 12 µL distilled water. A Biometra T-Gradient thermocycler was used with the following cycling conditions; 2 minutes pre-incubation at 94°C, followed by 34 cycles of 30 seconds at 94°C, 30 seconds at 55°C, 1 minute at 72°C, and final extension at 72°C for 2 minutes. The amplified PCR fragments were separated by electrophoresis through a 1.5% agarose gel in 1× TAE buffer at 100 Volts for 1 hour, prior to ethidium bromide staining and UV photography. PCR products of SR_SA_ and SR_NSW_ were purified and sequenced at the Australian Genome Research Facility, Brisbane. The resulting sequences were aligned with the previously sequenced RP5.11 amplified fragments of S_QLD_ and SR_QLD_, using the Clustalx program.

## Results

### Inheritance of resistance

#### Strong resistance in the New South Wales Strain (SR_NSW_) is encoded by more than one gene

**Figure 1:**
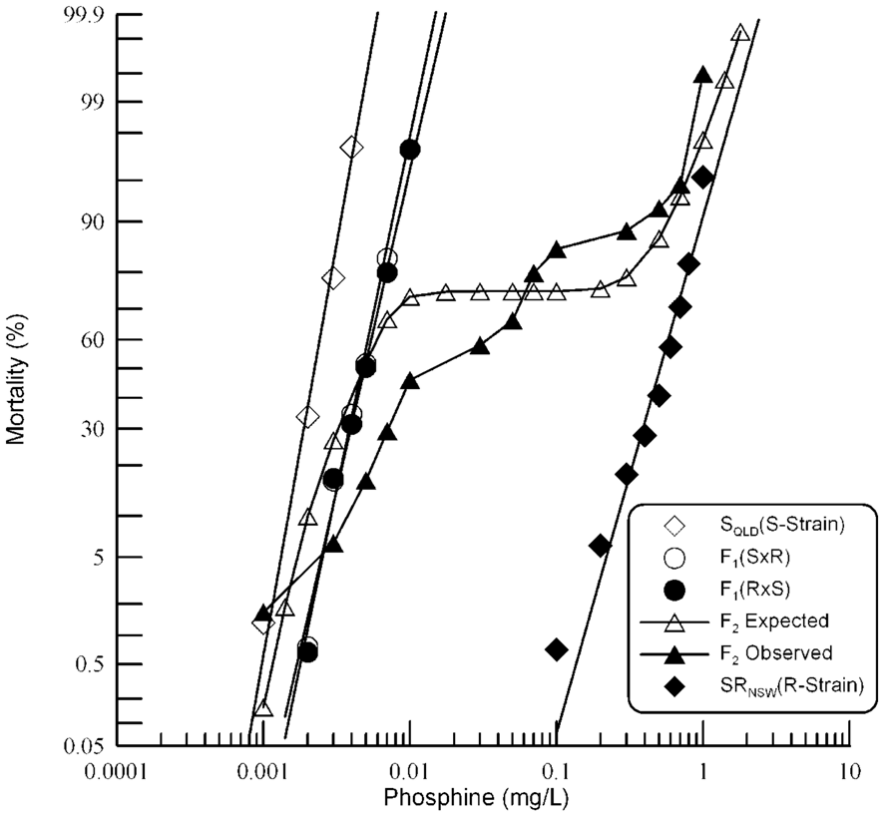
Response of F_1_ and F_2_ progeny of a cross between strongly resistant *R. dominica* from NSW and the susceptible reference strain to phosphine. Results are presented as log-dose mortality of the F_1_ hybrids and subsequent F_2_ progeny with reference curves of the parental strains, S_QLD_ (S-Strain) and SR_NSW_ (R-Strain). Phosphine exposure was for 48 hours at 25° and 55% r.h. The curve indicated by the open triangles is a hypothetical mortality curve for the F_2_ based on an assumption of resistance being conferred by a single recessive gene.

Probit analysis of mortality data for the sensitive (S_QLD_) and strongly resistant (SR_NSW_) parental strains and their reciprocal F_1_ progeny is presented in Table 1. Both S_QLD_ and the F_1_ progeny exhibited linear response curves (Fig. 1) indicating a homogeneous response to phosphine exposure. The resistant parental strain, on the other hand, exhibited a heterogenous response (heterogeneity factor: 3.42, χ^2^ p = 0.0012) (Table 1). The shallower slope of the probit regression line compared with that of the homogeneous sensitive parental strain and the F_1_ progeny (Fig. 1) also suggested a complex response to phosphine. The resistance factor for SR_NSW_ was estimated to be ∼225-fold. The response curves of reciprocal F_1_ crosses were nearly coincident (Fig. 1) and their LC_50_ values were not significantly different, as determined by overlap of their fiducial limits (Table 1). This absence of a maternal effect indicates that the resistance is autosomal. Therefore, the data from the reciprocal F_1_ crosses were pooled for subsequent statistical analyses. The mortality response of the pooled F_1_ was closer to that of the sensitive strain than the resistant strain with a degree of dominance −0.724 (±0.016) (where −1 = completely recessive and +1 = completely dominant), indicating an incompletely recessive expression of the resistance gene or genes. The resistance factor of 2.12 fold for the F_1_ with respect to the sensitive reference strain (S_QLD_) reflects the incompletely recessive nature of resistance.

**Table 1:** SR_NSW_×S_QLD_ - F_1_ probit analysis of phosphine sensitivity and test of strain heterogeneity.

*Strain (Cross)*	*n*	*Slope ± SE*	*LC_50_ (95% FL) (mg/L)*	*LC_99.9_ (mg/L)*	*df*	*χ^2^*	*P*
S_QLD_	2238	7.23±0.53	0.0023 (0.0021–0.0024)	0.0060	5	10.89	0.054
F_1_ (SxSR)	802	6.12±0.67	0.0047 (0.0043–0.0051)	0.015	4	7.74	0.102
F_1_ (SRxS)	712	5.59±0.32	0.0049 (0.0046–0.0051)	0.017	4	2.09	0.837
F_1_ (Pooled)	1542	5.85±0.42	0.0048 (0.0045–0.0050)	0.016	4	9.49	0.091
SR_NSW_	1557	4.51±0.39	0.51 (0.47–0.55)	2.5	8	23.97**	0.0012

Estimated lethal concentrations, slopes and goodness-of-fit tests of probit lines of the parental strains and their F_1_ progenies when insects were exposed to phosphine for 48 hours at 25°C and 55% r.h. SR_NSW_ = strongly resistant parental strain from New South Wales. S_QLD_ = phosphine sensitive parental strain from Queensland. F_1_ = first filial generation. n = number of individuals tested. SE = standard error. LC_50_ = the concentration at which 50% mortality is observed. LC_99.9_ = the concentration at which 99.9% mortality is estimated to occur. FL = fiducial limits. mg = milligrams. L = litre. df = degrees of freedom. *χ^2^* = chi-squared. P = probability value.

*Significant (P<0.05);

**Significant (P<0.01);

***Significant (P<0.001).

Observed mortality data from the F_2_ progeny were tested for goodness-of-fit to a hypothetical model of monogenic control of resistance. If phosphine resistance is controlled by a single gene, the phenotypes of F_2_ progenies are expected to be 25% sensitive and 75% resistant (if the resistance allele is dominant) or 75% sensitive and 25% resistant (if the resistance allele is recessive). As the F_1_ response data indicate that the resistance phenotype is nearly completely recessive, ∼75% of the progeny would be sensitive to phosphine if the trait was controlled by a single gene. This would manifest as a plateau in the F_2_ response curve at 75% mortality. However, no plateau was observed in this region of the curve (Fig. 1). Test of goodness-of-fit at individual doses (Table 2) indicated that the observed mortality was highly significantly different from the expected mortality at all but extremely low and high concentration as well as at the crossover point of ∼75% mortality. Hence, the hypothesis of monogenic control of resistance can be strongly rejected.

**Table 2:** SR_NSW_×S_QLD_ - F_2_ χ^2^ analysis of sensitivity to phosphine.

		Mortality				
Dose (mg/L)	n	Observed	Expected	Proportion surviving	χ^2^	P
0.001	149	3	0.2	98.3	22.07	<0.001
0.003	149	10	39.5	93.6	31.02***	<0.001
0.005	149	25	77.6	83.5	75.97***	<0.001
0.007	149	44	99.1	70.7	93.82***	<0.001
0.01	149	69	109.1	53.9	57.11***	<0.001
0.03	149	87	111.4	41.8	22.22***	<0.001
0.05	149	99	111.4	33.7	5.95**	0.015
0.07	149	119	111.4	20.2	1.82	0.177
0.1	149	127	111.4	14.8	8.19**	0.004
0.3	149	132	117.0	11.5	8.46**	0.004
0.5	149	137	129.5	8.1	2.94	0.087
0.7	149	141	138.7	5.4	0.35	0.552
1.0	198	197	193.5	0.5	2.83	0.093

Chi-squared analysis was carried out to determine whether mortality in an F_2_ population differed significantly from that expected if resistance was due to the effect of a single gene. Insects were exposed to phosphine for 48 hours at 25°C and 55% r.h. SR_NSW_ = strongly resistant parental strain from New South Wales. S_QLD_ = phosphine sensitive parental strain from Queensland. F_2_ = second filial generation. n = number of individuals tested. mg = milligrams. L = litre. *χ^2^* = chi-squared. P = probability value.

*Significant (P<0.05);

**Significant (P<0.01);

***Significant (P<0.001).

**Figure 2:**
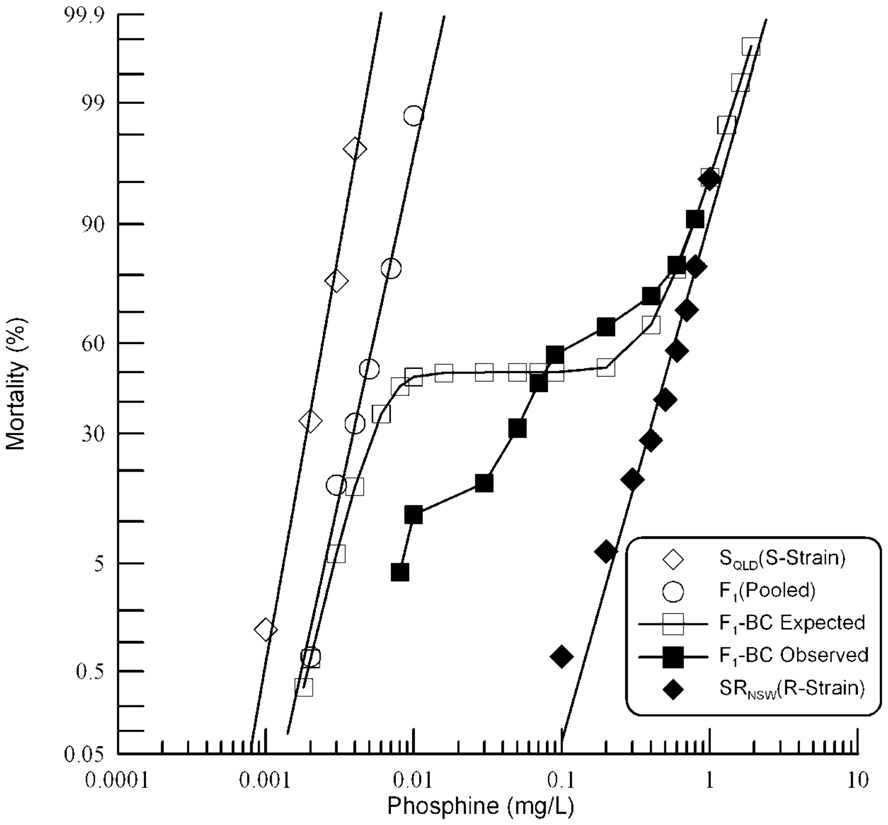
Resistance response of F_1_ hybrids and F_1_-BC progeny of a cross between strongly resistant *R. dominica* from NSW and the susceptible reference strain. Results are presented as log-dose mortality of the F_1_ hybrids and the F_1_-BC progeny with reference curves of the parental strains, S_QLD_ (S-Strain) and SR_NSW_ (R-Strain). Phosphine exposure was for 48 hours at 25° and 55% r.h. The curve indicated by the open square is a hypothetical mortality curve for the F_1_-BC based on an assumption of resistance being conferred by a single recessive gene.

Backcross analysis of the F_1_ progeny confirmed that resistance in SR_NSW_ is determined by more than one gene (Fig. 2). If a single gene is responsible for resistance in SR_NSW_, F_1_-BC progenies are expected to be 50% sensitive and 50% resistant, which will result in a plateau at ∼50% mortality on the F_1_-BC response curve. However, the observed F_1_-BC response (Fig. 2) indicated no plateau at this region. Chi-square analysis of the response to individual doses of phosphine (Table 3) revealed significant departure at all but extremely high concentrations, as well as the crossover point of the two curves at ∼50% mortality. There was a higher than expected mortality at high exposure and lower than expected mortality at low exposure as if genetic interactions or multiple additional genes with small effect are influencing mortality in those treatment ranges. As with the F_2_ analysis, the F_1_-BC data also support rejection of a model of monogenic resistance, suggesting that resistance in SR_NSW_ is controlled by two or more genes [39].

**Table 3:** SR_NSW_×S_QLD_ F_1_-backcross analysis of sensitivity to phosphine.

Dose (mg/L)	n	Mortality		Proportion surviving	χ^2^	P
		Observed	Expected			
0.006	117	0	42.1	100.0	65.78***	<0.001
0.008	117	5	53.0	95.7	79.37***	<0.001
0.01	117	13	56.7	88.9	65.47***	<0.001
0.03	117	20	58.5	82.91	50.67***	<0.001
0.05	117	37	58.5	68.4	15.80***	<0.001
0.07	117	56	60.5	53.6	0.57	0.448
0.09	121	73	65.0	43.9	5.41*	0.012
0.2	130	85	67.2	34.6	9.74**	0.002
0.4	130	97	85.9	25.4	4.25*	0.039
0.6	130	107	105.9	17.7	0.06	0.802
0.8	130	118	117.9	9.2	0.00	0.979
1.0	130	130	124.0	0.0	6.25*	0.012

Chi-squared analysis was carried out to determine whether mortality in an F_1_ backcross population differed significantly from that expected if resistance was due to the effect of a single gene. Insects were exposed to phosphine for 48 hours at 25°C and 55% r.h. SR_NSW_ = strongly resistant parental strain from New South Wales. S_QLD_ = phosphine sensitive parental strain from Queensland. F_1_ = first filial generation. n = number of individuals tested. mg = milligrams. L = litre. *χ^2^* = chi-squared. P = probability value.

*Significant (P<0.05);

**Significant (P<0.01);

***Significant (P<0.001).

#### Strong resistance in the South Australian Strain (SR_SA_) is encoded by more than one gene

**Figure 3:**
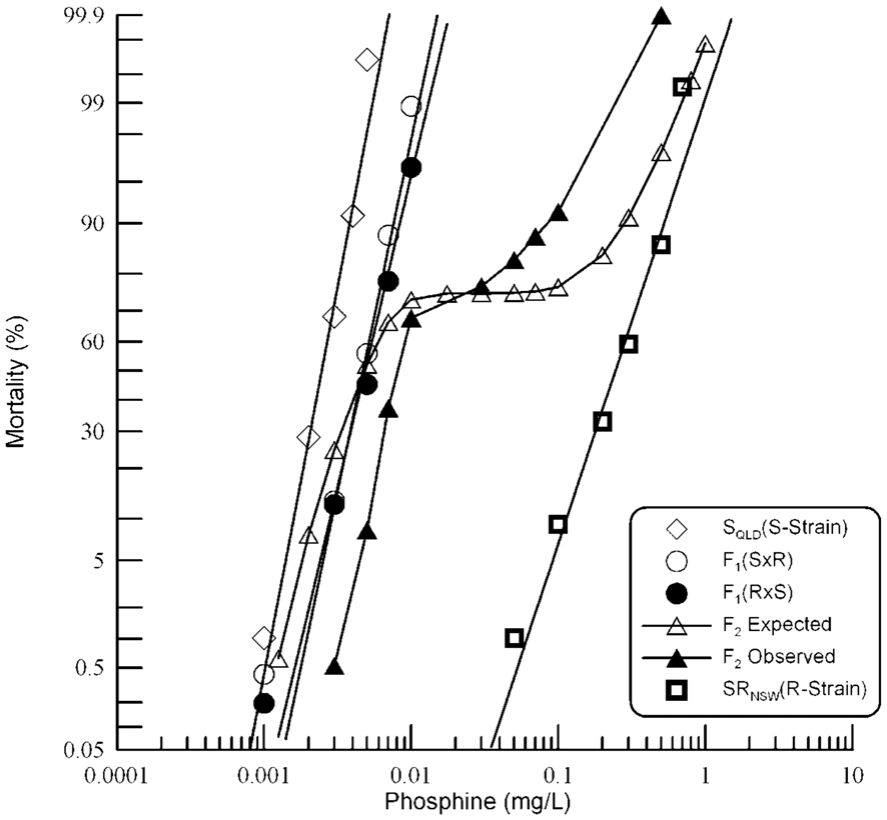
Resistance response of F_1_ and F_2_ progeny of a cross between strongly resistant *R. dominica* from South Australia and the susceptible reference strain. Results are presented as log-dose mortality of the F_1_ hybrids and subsequent F_2_ progeny with reference curves of the parental strains, S_QLD_ (S-Strain) and SR_SA_ (R-Strain). Log-dose mortality curve of the parental strains, S_QLD_ (S-Strain) and, their F_1_ hybrids and subsequent F_2_ progeny. Phosphine exposure was for 48 hours at 25° and 55% r.h. The curve indicated by the open triangles is a hypothetical mortality curve for the F_2_ based on an assumption of resistance being conferred by a single recessive gene.

Results of probit analysis of the parental strains and reciprocal F_1_ progeny from a cross between S_QLD_ and SR_SA_ are presented in Table 4. The response of the sensitive parental strain to phosphine was linear (Fig. 3) as was that of the reciprocal F_1_ crosses, indicating a homogeneous response. However, the response of the resistant parental strain was somewhat heterogeneous (heterogeneity factor: 3.73). The resistance factor of SR_SA_ following a 48 hour fumigation at 25°C was ∼100-fold, based on comparison of its LC_50_ value with that of S_QLD_. The LC_50_ values of reciprocal F_1_ crosses were not significantly different from each other, as determined by overlap of their 95% fiducial limits (Table 4). Similarly, the response curves of reciprocal F_1_ progeny were equivalent (Fig. 3.) The lack of a maternal effect indicates that the resistance is autosomally encoded. Because the progeny of the two crosses were phenotypically equivalent, data from the reciprocal F_1_ progeny were pooled for all subsequent analysis. Response of the pooled F_1_ progeny more closely resembled that of the sensitive strain than that of the resistant strain with a degree of dominance of −0.713 (±0.041). As the F_1_ progeny are only 1.94 times more resistant than the sensitive strain, the trait is nearly completely recessive.

**Table 4:** SR_SA_×S_QLD_ - F_1_ probit analysis of phosphine sensitivity and test of strain heterogeneity.

Strain/Cross	n	Slope ± SE	LC_50_ (95% FL) (mg/L)	LC_99.9_ (mg/L)	df	χ^2^	P
S_QLD_	2051	6.73±0.48	0.0025 (0.0023–0.0026)	0.0071	5	10.60	0.060
F_1_ (SxR)	512	6.07±0.52	0.0046 (0.0043–0.0049)	0.015	3	1.08	0.782
F_1_ (RxS)	455	5.43±0.33	0.0050 (0.0048–0.0053)	0.019	3	0.69	0.876
F_1_ (Pooled)	970	5.57±0.48	0.0048 (0.0044–0.0051)	0.017	3	1.52	0.678
SR_SA_	2058	3.87±0.34	0.24 (0.22–0.27)	1.5	6	22.35**	0.00101

Probit analysis used to determine strain heterogeneity and to estimate concentrations of phosphine required to achieve specific mortality endpoint. Parental strains and their reciprocal F_1_ progenies were exposed to phosphine for 48 hours at 25°C and 55% r.h. SR_SA_ = strongly resistant parental strain from South Australia. S_QLD_ = phosphine sensitive parental strain from Queensland. F_1_ = first filial generation. n = number of individuals tested. SE = standard error. LC_50_ = the concentration at which 50% mortality is observed. LC_99.9_ = the concentration at which 99.9% mortality is estimated to occur. FL = fiducial limits. mg = milligrams. L = litre. df = degrees of freedom. *χ^2^* = chi-squared. P = probability value.

*Significant (P<0.05);

**Significant (P<0.01);

***Significant (P<0.001).

A hypothetical model of monogenic control of resistance was tested using the F_2_ progeny. Since observation of the F_1_ response indicated that the resistance trait was almost completely recessive, the model predicts a plateau at ∼75% mortality. A shoulder is present in the curve at ∼75% mortality, but it is clear from the shape of the curve that it deviates significantly from the prediction based on a single gene model (Fig. 3). Test of goodness-of-fit of observed mortality data at individual doses to the mortality predicted by a single gene model (Table 5) revealed highly significant deviation at all but the lowest dose and the point of crossover at 75% mortality. The hypothesis of monogenic control of resistance was rejected on this basis. It seems probable, however, that while two or more genes contribute to resistance, one of them contributes disproportionately to the resistance phenotype.

**Table 5:** SR_SA_×S_QLD_ - F_2_ analysis of sensitivity to phosphine.

Dose (mg/L)	n	Mortality		Proportion surviving	χ^2^	P
		Observed	Expected			
0.001	191	0	0.3	100.0	0.305	0.581
0.003	191	1	46.0	99.5	58.024***	<0.001
0.005	191	16	99.2	91.6	145.193***	<0.001
0.007	191	71	126.7	62.8	72.841***	<0.001
0.010	239	162	174.9	32.3	3.674	0.055
0.030	239	195	179.3	23.0	5.528*	0.019
0.050	239	199	179.5	16.8	8.334**	0.004
0.070	239	210	180.7	12.2	19.208***	<0.001
0.100	239	219	184.4	8.4	28.178***	<0.001
0.500	239	239	232.1	0.0	6.803**	0.009
1.000	239	239	237.8	0.0	0.918	0.338

Chi-squared analysis was carried out to determine whether mortality in an F_2_ population derived from the F1 progeny of SR_SA_×S_QLD_ differed significantly from that expected if resistance was due to the effect of a single gene. Insects were exposed to phosphine for 48 hours at 25°C and 55% r.h. SR_SA_ = strongly resistant parental strain from South Australia. S_QLD_ = phosphine sensitive parental strain from Queensland. F_2_ = second filial generation. n = number of individuals tested. mg = milligrams. L = litre. *χ^2^* = chi-squared. P = probability value.

*Significant (P<0.05);

**Significant (P<0.01);

***Significant (P<0.001).

**Figure 4:**
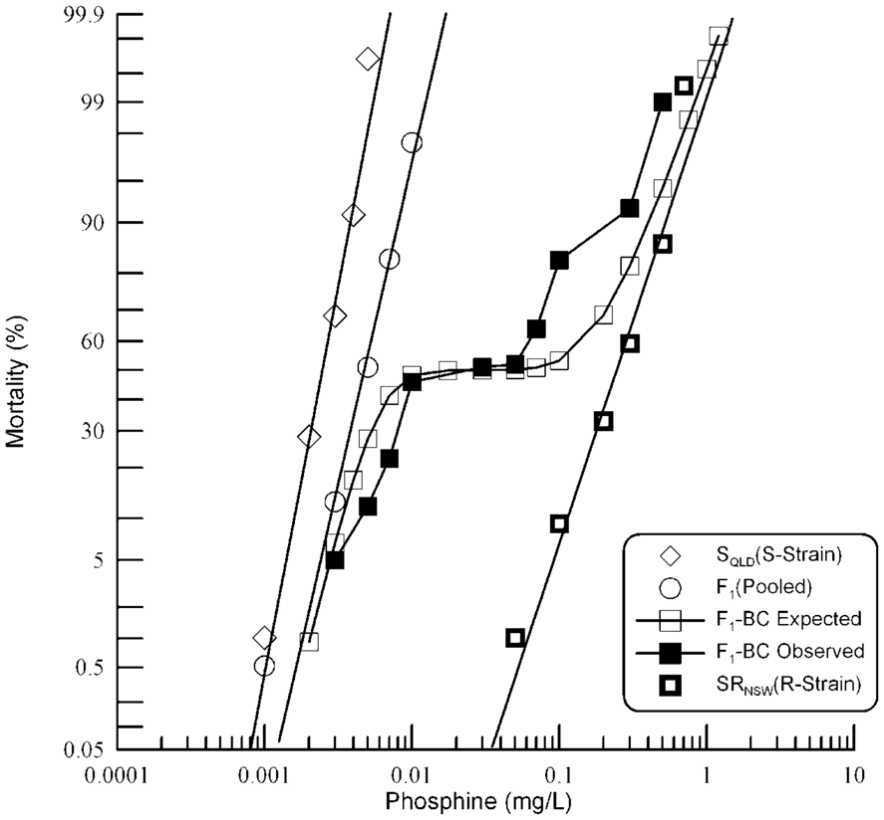
Resistance response of F_1_ hybrids and F_1_-BC progeny of a cross between strongly resistant *R. dominica* from South Australia and the susceptible reference strain. Results are presented as log-dose mortality of the F_1_ hybrids and the F_1_-BC progeny with reference curves of the parental strains, S_QLD_ (S-Strain) and SR_SA_ (R-Strain). Phosphine exposure was for 48 hours at 25° and 55% r.h. The curve indicated by the open square is a hypothetical mortality curve for the F_1_-BC based on an assumption of resistance being conferred by a single recessive gene.

The findings of the F_2_ analysis were then confirmed using a backcross progeny derived from mating a virgin female F_1_ beetle with its genetically recessive, phenotypically resistant male parent. If a single gene is responsible for resistance in SR_SA_, the response curve of the F_1_-BC was expected to plateau at 50% mortality. Visual inspection of the F_1_-BC response curve (Fig. 4) reveals a significant plateau in the predicted mortality range, suggestive of monogenic resistance. However, the plateau is unexpectedly narrow and the mortality curve deviates from the prediction, particularly for beetles exposed to high concentrations of phosphine. When the Chi-square test was applied to F_1_-BC data for individual doses of phosphine (Table 6) significant congruence with the single gene model was observed at ∼50% mortality, which corresponds to the plateau of the curve. Other than that, the values deviated significantly for all but the lowest and highest doses. These results are consistent with more than one gene being responsible for resistance, though one of the genes would seem to disproportionately contribute to the resistance phenotype [39]. Despite a degree of similarity with the expectations of a single gene model of resistance, both the F_2_ and F_1_-BC data deviate significantly from the expectations of the model, indicating that monogenic inheritance is not able to explain the phosphine resistance of the strongly resistant South Australian strain, SR_SA_.

**Table 6:** SR_SA_×S_QLD_ - F_1_-backcross analysis of sensitivity to phosphine.

Dose (mg/L)	n	Mortality		Proportion surviving	χ^2^	P
		Observed	Expected			
0.003	60	3	4.1	95.0	0.294	0.588
0.005	75	9	20.7	88.0	9.183**	0.002
0.007	85	19	35.2	77.7	12.725***	<0.001
0.01	100	46	48.2	54.0	0.201	0.65
0.03	100	51	50.0	49.0	0.039	0.843
0.05	100	52	50.2	48.0	0.131	0.718
0.07	100	64	50.9	36.0	6.872**	0.008
0.1	100	83	53.3	17.0	35.346***	<0.001
0.3	100	92	81.8	8.0	7.02**	0.006
0.5	100	99	94.3	1.0	4.115*	0.043
1	100	100	99.6	0.0	0.447	0.504

Chi-squared analysis was carried out to determine whether mortality in an F_1_ backcross population differed significantly from that expected if resistance was due to the effect of a single gene. Insects were exposed to phosphine for 48 hours at 25°C and 55% r.h. SR_SA_ = strongly resistant parental strain from South Australia. S_QLD_ = phosphine sensitive parental strain from Queensland. F_1_ = first filial generation. n = number of individuals tested. mg = milligrams. L = litre. *χ^2^* = chi-squared. P = probability value.

*Significant (P<0.05);

**Significant (P<0.01);

***Significant (P<0.001).

### Complementation Analysis

Two genes, *rph1* and *rph2*, are responsible for the phosphine resistance phenotype in a strongly resistant strain of *R. dominica* from Queensland, Australia [11]. We carried out complementation analysis using a strain carrying the resistance allele of *rph1* to determine whether this gene also contributes to resistance in SR_NSW_ and SR_SA_.

#### The rph1 gene previously identified in WR_QLD_, contributes to resistance in SR_NSW_

**Figure 5:**
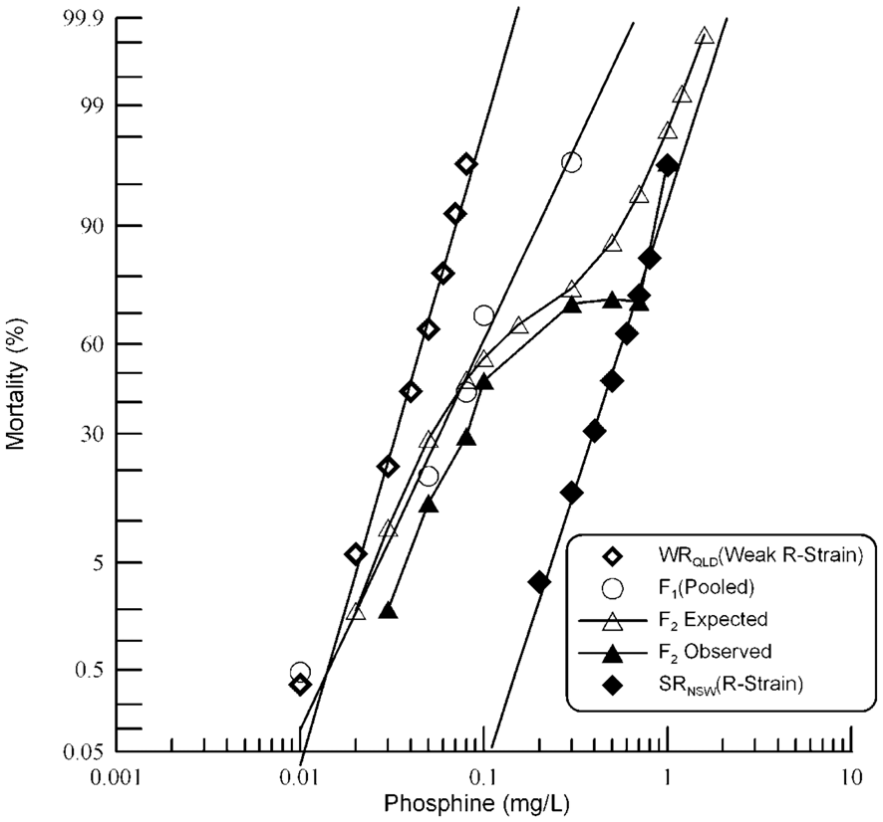
Resistance response of F_1_ hybrids and F_2_ progeny of a cross between strongly resistant *R. dominica* from NSW and the weak resistant strain from Queensland. Results are presented as log-dose mortality of the F_1_ hybrids and the F_2_ progeny with reference curves of the parental strains, WR_QLD_ (Weak R-Strain) and SR_NSW_ (R-Strain). Phosphine exposure was for 48 hours at 25° and 55% r.h. The curve indicated by the open triangle is a hypothetical mortality curve for the F_2_ based on an assumption of resistance being conferred by a single recessive gene.

The strongly resistant strain SR_NSW_ was crossed with the weakly resistant strain from Queensland (WR_QLD_), which is homozygous for the resistance allele of the *rph1* gene. Probit analysis of the response data of parental strains WR_QLD_ and SR_NSW_ as well as their F_1_ progeny revealed liner response curves (Fig. 5) indicating that each population tested exhibited a homogeneous response. Chi-square analysis of the response data also suggested that the populations were genetically uniform, with the following Chi-square values: SR_NSW_, 8.239 (df = 7, p = 0.312); WR_QLD_, 4.95 (df = 7, p = 0.666); and for the F_1_, 7.03 (df = 3, p = 0.071).

If the *rph1* gene contributes to resistance in both SR_NSW_ and WR_QLD_, the F_1_ progeny of the cross would be expected to exhibit a resistance phenotype at least as strong as the weakly resistant strain, WR_QLD_. If this gene does not contribute to resistance in SR_NSW_, the strains would fully complement each other, resulting in F_1_ offspring that were nearly completely sensitive to phosphine. The observed response curve of the F_1_ progeny demonstrates that the hybrids are slightly more resistant to phosphine than is WR_QLD_, as would be expected if both parental strains contain a resistance allele at *rph1*. An additional, incompletely recessive resistance allele in SR_NSW_ could explain the slightly stronger resistance of the hybrids relative to WR_QLD_ (Fig. 5).

The F_2_ progeny were then analysed to determine how many resistance genes exist in SR_NSW_, in addition to *rph1*, which is shared by the two parental strains. A plateau in the response curve was observed at ∼75% mortality (Fig. 5), clearly indicating that a single gene, in addition to *rph1*, is responsible for the strong resistance phenotype of SR_NSW_. The unusual resistance observed at lower doses of phosphine exposure, previously noted in the F_2_ progeny of the cross between SR_NSW_ and S_QLD_, is also apparent in the F_2_ progeny of the cross between SR_NSW_ and WR_QLD_. While the cause of this phenotype is unclear, it seems to be a feature of the SR_NSW_ strain rather than of S_QLD_ or WR_QLD_ or the *rph1* gene itself.

#### The rph1 gene also contributes to resistance in SR_SA_

**Figure 6:**
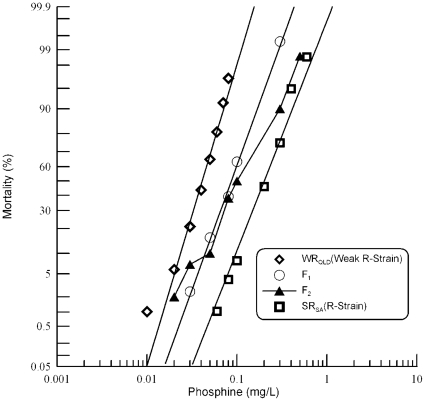
Resistance response of F_1_ hybrids and F_2_ progeny of a cross between strongly resistant *R. dominica* from SA and the weak resistant strain from Queensland. Results are presented as log-dose mortality of the F_1_ hybrids and the F_2_ progeny with reference curves of the parental strains, WR_QLD_ (Weak R-Strain) and SR_SA_ (R-Strain). Phosphine exposure was for 48 hours at 25° and 55% r.h.

As with SR_NSW_, the strongly resistant strain from South Australia, SR_SA_ was also crossed to WR_QLD_, which is homozygous for the resistance allele at *rph1*. Responses of both SR_SA_ and WR_QLD_, as well as their F_1_ progeny revealed liner response curves (Fig. 6) suggesting that the strains are genetically homogeneous. As with SR_NSW_, the F_1_ progeny were slightly more resistant to phosphine than was WR_QLD_, which indicates that, just like SR_NSW_, a resistance allele in *rph1* also contributes to resistance in SR_SA_. Interestingly, the partial dominance exhibited by a putative second resistance gene in SR_SA_ is equivalent to that observed previously in SR_NSW_, despite the fact that, when homozygous, the resistance factor in SR_NSW_ is much greater than in SR_SA_.

The response curve of the F_2_ progeny gives only a hint of a plateau at 75% mortality. This is likely due to the relatively small contribution of the second resistance factor to the resistance phenotype as the strong resistance of SR_SA_ is much lower than that of either SR_NSW_ or SR_QLD_ [11]. There is simply not much difference in the level of resistance between individuals heterozygous for a second resistance factor and those homozygous for a second resistance factor in a genetic background of homozygosity for *rph1*. It is striking, therefore, that the semi dominance of the second resistance factor is equivalent in all three strains. On balance, we can say that the phosphine resistance phenotype in SR_SA_ is conferred by a resistance allele at *rph1* as well as by at least one other resistance factor.

Whereas the F_2_ progeny of crosses involving SR_NSW_ are unusually resistant at low dose exposure to phosphine, F_2_ progeny of crosses involving SR_SA_ show normal resistance at low doses, but unexpected sensitivity at high doses. This is most clearly seen in Figure 3 but also seems to be the case in Figure 6. The causes of these unique mortality characteristics are unclear.

### Molecular Diagnostic of Phosphine Resistance

**Figure 7:**
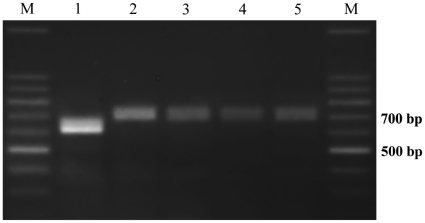
Polymorphic PCR fragments amplified by specific primers RP5.11 in five strains of *R. dominica*: SR_QLD_ (1), S_QLD_(2), WR_QLD_(3), SR_NSW_(4), SR_SA_(5), M(100 bp DNA ladder). The short fragment polymorphism is unique to SR_QLD_.

A pair of primers specific to the *rph2* locus (STS5.11) was used to determine whether a polymorphism previously found linked to *rph2* in SR_QLD_ was similarly found in SR_NSW_ and/or SR_SA_. PCR using DNA extracted from each strain produced one of two alternative fragments (Fig. 7). The STS5.11 fragments amplified from SR_NSW_ and SR_SA_ were sequenced and found to be 673 bp in length and identical in sequence to that of S_QLD_ [12] (Genbank accession GF111941). In contrast, the sequence of SR_QLD_ (Genbank accession GF111942) lacks an 80 nucleotide sequence present in the other three strains, but is otherwise identical to the other sequences. This result indicates that the strongly resistant *R. dominica* in New South Wales and South Australia did not simply originate from strongly resistant insects transported from Queensland.

## Discussion

The world-wide reliance on phosphine for the protection of stored commodities from insects makes it extremely significant both economically and in terms of global food security. The disproportionate reliance on a single chemical makes understanding the evolution of resistance of paramount importance. Because of the unique toxicological properties of this fumigant, and its method of application, we cannot simply extrapolate from our understanding of resistance to contact pesticides in field pests and assume that we understand the selection process that leads to insects that are resistant to the fumigant, phosphine. The Australian situation provides a compelling research opportunity as phosphine resistance has been monitored for more than two decades and genetic analysis of the resistance is well advanced [10]–[12], [40]. This situation can be used to understand the evolution of resistance, which can then be applied to the global problem.

The current work refines our understanding of how resistance originates and supports a recently proposed model of constraints on the evolution of resistance [12]. We have achieved this by comparing the genetics of strongly resistant *R. dominica* strains from New South Wales (SR_NSW_) and South Australia (SR_SA_), to a previously described strongly resistant strain (SR_QLD_), isolated from Queensland, Australia in 1997 [6]. Interpretation of these results relies on previous molecular studies [11], [12], which determined that strong resistance in SR_QLD_ (referred to as QRD569) resulted from the sequential acquisition of resistance alleles at two major loci.

The strength of the resistance phenotype in all of the resistant strains was determined relative to a fully sensitive reference strain from Queensland, S_QLD_ (referred to as QRD14) that has been employed in our previous analysis of phosphine resistance in Queensland. We determined the resistance factor of the New South Wales strain, SR_NSW_, to be ∼225 times that of the sensitive reference strain, whereas the resistance factor for the South Australian strain, SR_SA_, was only ∼100 times. These levels of resistance were somewhat less than the resistance factor reported for the strongly resistant strain from Queensland, SR_QLD_, of ∼600-fold [10], though in each strain, resistance is much greater than a simple additive effect of the two resistance loci. The three strongly resistant *R. dominica* strains originated from widely separated geographical locations of Australia, many hundreds of kilometres apart from each other. Evidence that they are not only geographically distinct, but genetically distinct as well comes from a molecular marker linked to resistance in SR_QLD_ that is absent in the other two strains. The fact that the phenotypic level of resistance in each strain is also quite distinct, particularly that of SR_SA_, provides additional support for the notion that the strains are of distinct origins.

Reciprocal crosses between each of the two strongly resistant strains and the sensitive reference strain resulted in F_1_ progeny that displayed equivalent resistance regardless of the sex of the resistant parent. The absence of maternal inheritance of resistance clearly demonstrates that the resistance factor is not mitochondrially inherited. This is significant in light of the proposed mitochondrial target of phosphine [41]. The result was not unexpected, however, as a lack of mitochondrial inheritance had previously been demonstrated for SR_QLD_ [10] and for phosphine resistance in other strains of insects as well [7], [40], [42]. The lack of sex linkage as indicated by the uniformity of the mortality response in the reciprocal F_1_ populations, indicates that the trait is encoded on an autosomal chromosome.

Resistance of F_1_ progeny resulting from crosses between the sensitive strain S_QLD_ and either SR_NSW_ or SR_SA_, was closer to that of the sensitive parental strain S_QLD_ than to the respective resistant parent. Thus, the trait is incompletely recessive with the resistance factor of the hybrid progeny of both crosses ∼2 times the resistance level of the sensitive parent. It is interesting that the resistance attributable to the trait being incompletely recessive is the same between the two strains, whereas when the parental strains are homozygous resistant, there is a 2 fold difference between them. The well studied strain from Queensland, SR_QLD_, exhibited a similar level of semi-dominance [10], most of which was attributable to *rph2* [12], one of two genes responsible for strong resistance.

Both SR_NSW_ and SR_SA_ resemble previously studied strains of phosphine resistant *R. dominica* from Australia [10] and the Philippines [7], in which two or more major factors are responsible for the strong resistance phenotype. Very few genetic studies of phosphine resistance have been carried out on insect species other than *R. dominica*. Strong resistance to phosphine in *Sitophylus oryzae* (L.) is also controlled by two or more genes [42]. In the red flour beetle, *Tribolium castaneum* (Herbst), a single major gene contributes to a weak resistance phenotype [40]. The response of the F_1_-BC progeny of S_QLD_×SR_NSW_ clearly indicates that at least two major genes are responsible for the resistance phenotype. Interpretation of the equivalent backcross progeny of S_QLD_×SR_SA_, is not so straightforward. In the case of SR_SA_, it seems that one major resistance gene exists with the resistance allele of a second gene being relatively weak. We cannot dismiss the possibility that additional minor genes contribute to resistance, though, at least in the case of SR_NSW_, any such effect is masked by the strength of the two primary resistance genes.

The fact that two genes are found to contribute to high-level resistance in multiple cases does not imply that the same two genes are involved in each instance. Knowing whether this is the case has profound practical implications for resistance management. For example, expensive and disruptive quarantine measures might be warranted only if there is a risk of various resistance genes combining to create extremely high levels of resistance. In contrast, if there are few ways that resistance can evolve, it may be possible to devise effective strategies to slow the development of resistance in regions where resistance does not yet exist.

Weak resistance to phosphine is ubiquitous across the grain growing regions in Australia. It is therefore possible to study strains from widely separated regions to determine whether the same genes confer resistance in each instance. Indeed, we found that the resistance trait in both SR_NSW_ and SR_SA_ is partially due to a resistance allele at *rph1*, a gene previously found to contribute to resistance in SR_QLD_. It is interesting to note that the level of resistance contributed by the allele at *rph1* seems to be equivalent in all three strains. Two possible explanations exist for this situation. Either, a single mutational event occurred and proliferated due to a selective advantage and spread across the grain-growing regions through transport of grain or migration of insects. Alternatively, independent mutations occurred in the same target gene in each resistance outbreak. Grain transport in Australia is predominantly by rail, directly from farm to port, with relatively little lateral transfer of grain between regions. Thus, it is unlikely that outbreaks of highly resistant beetles in southeast Queensland, central New South Wales and South Australia originated from a single mutation that subsequently spread. Precedence for the second model, in which mutations occur independently in a single resistance gene comes from studies of dieldrin resistance, in which resistant insects of many species in many countries originated due to independent mutations in a single target gene, *Rdl* [43]–[45].

### Concluding remarks

Given the real threat posed by insect pests that are highly resistant toward phosphine, it is easy to lose sight of the likelihood that phosphine will be used for decades to come. The fact that the resistance genes are almost completely recessive and only confer high level resistance in combination dictates that the strong resistance phenotype depends on homozygosity at both loci and will therefore be expressed as the product of the square of the frequency of each allele in the population. Thus SR = *rph1* ^2^×*rph2* ^2^ in which SR is the frequency of the strong resistance phenotype and *rph1* and *rph2* are the frequencies of their respective resistance alleles in the population. This relationship highlights the fact that the effective population of insects that are likely to survive fumigation by virtue of a strong resistance genotype will be much lower than the actual population of insects that carry resistance alleles. This advantage only exists, however, while the frequencies of the resistance alleles are low. Given this scenario, fumigation practices that allow survival of weakly resistant insects in regions that have previously experienced high level resistance outbreaks are likely to result in the rapid development of ubiquitous and abundant high level resistance. Thus, fumigating grain below the recommended rate in an effort to save money, as well as repeated fumigations of the same bulk of grain, have to be considered risky activities with the potential to seriously damage the prospect of long-term phosphine usage.
